# A Study on the Job Characteristics of Police Officers and the Status of Musculoskeletal Disorders

**DOI:** 10.3390/healthcare12191983

**Published:** 2024-10-04

**Authors:** Wonnam Kim, Seohyun Park, Byeong Kwan Woo, Yeonhak Kim, Changwoog Yoon, Dongmin Lee, Jion Kim, Yeon-Cheol Park

**Affiliations:** 1 Division of Pharmacology, School of Korean Medicine, Pusan National University, Yangsan 50612, Republic of Korea; eb75lab@pusan.ac.kr; 2 Department of Clinical Korean Medicine, Graduate School, Kyung Hee University, Seoul 02453, Republic of Korea; smart819@khu.ac.kr (S.P.); minya21c@khu.ac.kr (D.L.); 3 School of Police Administration, Seowon University, Cheongju 28644, Republic of Korea; byeongkwan.woo@seowon.ac.kr; 4 Department of Korean Medicine, School of Korean Medicine, Pusan National University, Yangsan 50612, Republic of Korea; kimyh930@pusan.ac.kr; 5 Department of Computer Science, College of Natural Sciences, Korea National Open University, Seoul 03087, Republic of Korea; dkdi4236@knou.ac.kr; 6 Department of Forensic Information Science and Technology, Hallym University, Chuncheon 24252, Republic of Korea; 7 Department of Acupuncture & Moxibustion, Kyung Hee University College of Korean Medicine, Kyung Hee University Hospital at Gangdong, Seoul 05278, Republic of Korea; 8 Department of Acupuncture and Moxibustion, College of Korean Medicine, Kyung Hee University, Seoul 02453, Republic of Korea

**Keywords:** police officers, musculoskeletal disorders, survey research, shoulder pain, lower back pain

## Abstract

Musculoskeletal disorders occur frequently in industrial settings, and police officers in particular are at high risk of developing musculoskeletal disorders due to the nature of their work, but research on this is lacking. Therefore, the purpose of this study was to examine the prevalence and risk factors of musculoskeletal disorders according to the job functions of police officers. A survey was conducted, targeting active-duty police officers, and data were collected from 4268 respondents who answered all items on the questionnaire. As a control group, we used the general population dataset from the 2020 National Health and Nutrition Examination Survey conducted in Korea. The survey included general information such as gender, age, working hours, and type of work. It also investigated medical utilization, including the frequency of medical visits and hospitalizations. The assessment of musculoskeletal disorders was analyzed using pain NRS, VAS, SPADI, and ODI. The working characteristics of police officers were found to be more irregular in terms of working hours and involved a higher intensity of work activities compared with the general population. However, there was a lack of precise diagnoses and continuous treatment for these disorders. These results imply that police officers’ occupational characteristics are related to musculoskeletal disorders. Considering the direct relationship between police officers’ health and public safety, systematic evaluation and management of their musculoskeletal disorders are needed.

## 1. Introduction

Police officers’ core responsibilities are closely linked to safeguarding the lives and property of citizens. Consequently, police officers’ health and safety are highly correlated with the public’s well-being and safety. Police officers are at a higher risk of developing various health conditions, including musculoskeletal disorders (MSD), post-traumatic stress disorder (PTSD), cardiovascular diseases, and cerebrovascular diseases, due to the prolonged wearing of various equipment (firearms, handcuffs, etc.), frequent irregular night shifts, and the risk of physical injuries [1]. According to a health-related research report conducted by the Korean Metropolitan Police in 2015, police officers have a higher risk of cardiovascular and cerebrovascular diseases than administrative government officials. In particular, it was reported that the risk of ischemic heart disease increases twofold, and the risk of myocardial infarction increases fourfold [2]. Furthermore, the prevalence of PTSD among police officers has been consistently increasing [3], and they have a higher risk of shoulder disorders than firefighters [4]. Additionally, police officers reportedly have higher rates of various cancers, including blood cancer, brain cancer, liver cancer, lung cancer, stomach cancer, colon cancer, and bladder cancer, compared with other occupational groups. The increased risk of these conditions among police officers is attributed to factors such as frequent night shifts, rotating shifts, wearing equipment, and frequent exposure to traumatic events [5]. Among these factors, the correlation between night shift work and cardiovascular and cerebrovascular diseases has been confirmed through numerous studies [6]. Frequent exposure to traumatic incidents during their duties can impact police officers, leading to PTSD [7], and factors such as prolonged wearing of equipment [8] and extended periods of muscle tension due to activities such as prolonged motorcycle driving [9] can influence musculoskeletal disorders. Moreover, tinnitus and hearing impairment increases because of tasks related to radios and public demonstrations [10].

Specifically, factors such as wearing police gear (firearms and radios), apprehending suspects in the act, prolonged driving of police vehicles, extended hours of investigative work, encounters with protesting crowds, intensive crowd control training, wearing protective gear during protests, directing traffic, and extended periods of motorcycle patrol duty have been investigated as factors that exacerbate musculoskeletal disorders. In particular, equipment use has been confirmed to result in high levels of physical fatigue [11]. Additionally, irregular shift work can disrupt the body’s natural rhythm and cause excessive muscle tension, further worsening general health [12].

According to the 2021 South Korean industrial accident statistics, there were 122,713 industrial accident victims (accident rate = 0.63%) who required treatment for more than 4 days (2080 deaths, 101,182 injuries, and 19,183 occupational disease patients), and among those who received treatment for occupational diseases, 11,868 cases (61.86%) were related to musculoskeletal diseases. Of these, 6549 cases (55.18%) occurred in physically demanding work [13]. Various studies on work-related musculoskeletal disorders (WMSD) have investigated the prevalence, vulnerable body parts, and aggravating factors by occupation. Studies have shown that healthcare workers were most susceptible to WMSD in the back, neck, shoulders and wrist areas. Surgeons and dentists had the highest prevalence of MSDs in the lumbar spine (60%), shoulder, and upper extremities (33–55%); nurses had the highest prevalence of MSD in the lower extremities (≥25%) [14]; and physical therapists had the highest prevalence of MSD in the lower back (40.1%), thumb (35.4%), neck (17.7%), and shoulder (20.8%) [15]. Other studies have shown that 20–60% of general office workers suffer from MSD related to the upper limbs and neck [16], and the main risk factor for developing WMSD is excessive and repetitive physical work [17]. This indicates that work-related musculoskeletal disorders occur frequently in industrial settings, and police officers in particular are more likely to suffer from musculoskeletal disorders due to the nature of their work. However, there is a lack of research in Korea on the musculoskeletal disorders caused by police work. In foreign countries, the public liability estimation system, including for police officers and other government officials, is well established and effectively applied. In the United States, the Federal Employees’ Compensation Act came into effect, providing compensation to federal employees, including police officers. In California, there is a specific provision known as the “duty belt presumption” under the workers’ compensation system for musculoskeletal disorders caused by wearing duty belts during work, offering clear guidelines for compensation for police officers experiencing pain in the lower back and below. However, in Korea, there is no presumed system for police officers regarding musculoskeletal disorders, and there is a notable shortage of investigations or research focusing on musculoskeletal disorders.

Therefore, the purpose of this study was to investigate the symptoms of musculoskeletal disorders and their job-relatedness through a survey targeting current police officers. The results can be used as basic data for the development of various programs and policies to promote the health of police officers.

## 2. Materials and Methods

The research process involved the formation of a consensus group comprising researchers, medical experts specializing in musculoskeletal disorders, active-duty police officers, the general public, survey experts, and statisticians. This group collaborated to develop survey questionnaires and a research plan aimed at analyzing job-related factors related to musculoskeletal disorders in police officers, proposing the presumption criteria, and deriving health management strategies. Ethical approval for this research related to the survey was obtained from the Institutional Review Board of Kangdong Kyung Hee University Hospital (KHNMCOH 2022-08-006-004). All procedures of the survey study were conducted by researchers in accordance with the Declaration of Helsinki and Korean clinical trial practice guidelines.

This study was registered on the SurveyMonkey system, and from 1 October 2022 to 15 November 2022, electronic emails or text messages containing explanations about the survey and a URL link to the survey questionnaire were sent to all police officers through the Korean National Police Agency. Police officers who voluntarily agreed to participate in the study and responded to the survey were included in the analysis. In total, 9422 police officers participated in the survey, of whom 4268 responded to all survey items. For comparison, we used data from the 2020 Korean National Health and Nutrition Examination Survey, which included 2533 individuals.

Separate datasets were generated for police officers, excluding those who did not respond to the survey, those who did not disclose their sex, or those who did not disclose their age. Similarly, a public dataset was created using data from the 2020 National Health and Nutrition Examination Survey. Subsequently, value-matching between the police officer and general public datasets was conducted. The matched datasets for police officers and the general public were then integrated for further analysis.

The survey included general information (sex, age, household composition, working hours, and work type), healthcare contacts (hospitalization, outpatient visits and frequency, reasons for not seeking outpatient care, and additional examinations), and physical activity (occupational physical activity and duration, leisure-related physical activity and duration, and strength exercises). These items were designed to match Korea’s 2020 National Health and Nutrition Examination Survey for a comparative evaluation between police officers and the general population [18]. The assessment of musculoskeletal disorders involved an investigation and analysis of pain intensity, the shoulder pain and disability scale (SPADI) [19], and the lumbar pain and disability scale (Oswestry Disability Index, ODI) [20].

The survey comprised 153 questions organized into seven categories covering general health information, physical activity, and assessment of musculoskeletal disorders. The general health information section contained 23 questions. Among these questions, those on sex, age, household composition, average weekly working hours, and work type were identical to those in the 2020 National Health and Nutrition Examination Survey. Work type included inquiries about average weekly working hours, primary working hours, and shift work types, including overtime/night shifts.

The section on physical activity comprised 15 questions aligned with the 2020 National Health and Nutrition Examination Survey questions. It included inquiries about occupation-related physical activity and duration (high and moderate intensity), leisure-related physical activity and duration (high and moderate intensity), and strength training. High-intensity physical activity involves pursuits such as chasing suspects, making arrests, physical altercations with intoxicated individuals, and engaging in physical confrontations during protests or demonstrations. Moderate-intensity physical activity encompasses activities related to accompanying and protecting intoxicated individuals, using physical restraints as safety measures, foot patrols, and conducting physical searches during criminal investigations. As a standard for high-intensity exercise and leisure activities, it was described as an activity in which the person was continuously out of breath for at least 10 min or the heartbeat was very fast. The criteria for medium-intensity exercise and leisure activities were presented as activities in which the person was slightly out of breath for at least 10 min or the heartbeat was slightly faster. Strength training questions inquired about the number of days in the past week in which individuals had engaged in activities such as walking for at least 10 min and performing exercises such as push-ups, sit-ups, or weightlifting. The musculoskeletal questionnaire consisted of 115 questions that focused on discomfort and symptoms related to musculoskeletal issues resulting from continuous or repetitive work activities. It consisted of experience with repetitive shoulder and back use in the work environment, and the frequency and intensity of repetitive use. Shoulder and lumbar pain were evaluated for pain intensity using the National Institute of Occupational Safety and Health (NIOSH) criteria, and the shoulder was examined for range of motion. The SPADI and ODI were used to assess the severity of shoulder and lumbar pain and disability, respectively.

The visual analog scale (VAS) was used to assess pain intensity. During the survey, the participants directly marked a mobile pain scale, where 0 represented “no pain at all”, and 100 indicated “unbearable severe pain”. The VAS is a visual analog rating scale ranging from 0 to 100, with 0 representing no pain and 100 representing intolerably severe pain [21]. Pain intensity exceeding 50 points on the VAS may indicate the need for specialized medical attention. The VAS is a widely used assessment tool for various types of pain, including acute abdominal pain [8], and has demonstrated reliability in evaluating patients with chronic musculoskeletal pain [22].

The SPADI was used to evaluate shoulder pain and function. During the survey, participants rated their responses on a scale ranging from 0 (no pain or difficulty) to 10 (severe pain or inability to perform duties without assistance) for each item. The SPADI was developed by Roach (1991) to assess shoulder pain and function. The questionnaire is widely used [19] and has proven reliability and validity for assessing the functional impairment of the shoulder [23]. In various domestic studies, the SPADI has been used as an assessment tool for shoulder pain. The questionnaire consists of five items for the pain subscale and eight items for the function/disability subscale, totaling 13 questions, all rated on a VAS [24]. The higher the SPADI score, the more severe the level of pain and disability.

The ODI was used to assess lower back pain and function. The ODI is a lower back pain indicator consisting of 10 evaluations related to the movements of daily life [20]. The evaluation items include pain intensity, personal care, lifting, walking, sitting, standing, sleeping, sex life, social life, and travel. Each item is rated on a scale of 0–5 in six steps. The results are calculated as percentages, with 0% indicating no functional disability and 100% representing the highest level of functional disability. The ODI is widely used as an indicator of lower back pain and is known for its high reliability [25]. Depending on the study subjects, there may be variations; however, a score of 10–12 points is generally considered to indicate minimal clinical significance. Many cases of lower back pain with accompanying functional disability have a score of 12 points or higher. In general, 10 points represent a typical case, 10–20 points indicate lower back pain without disability, and scores of >20 points signify lower back pain with functional impairment [26].

To assess musculoskeletal symptoms and risk factors, a survey was conducted, based on the criteria provided by the NIOSH, which established a 3-stage criterion for symptoms of musculoskeletal disorders. The first stage involves symptoms persisting for more than one week, with mild pain occurring at least once a month. The second stage involves symptoms persisting for more than one week, with moderate pain occurring at least once a month. The third stage involves symptoms persisting for more than one week, with severe pain occurring at least once a month [27].

General information for the general public and police officers was described using descriptive statistics. Categorical data were presented as frequencies and compared using chi-square tests, and continuous variables were presented as the mean and standard deviation and compared using independent t-tests. To compare physical activity, each item was expressed as the frequency and proportion, and the analysis was performed using chi-square tests. The results were expressed as OR and 95% CI. A *p* value < 0.05 was considered statistically significant. To examine the characteristics of police officers complaining of shoulder pain, the number and proportion of subjects exposed to aggravating factors were described, and the frequency, proportion, and intensity (VAS) of the occurrence of pain by factor were investigated. In addition, police officers who complained of shoulder pain within the past month or year were divided into four groups, the frequency and proportion were indicated, and shoulder pain and function were evaluated using the SPADI. The mean and standard deviation were described by group, and the comparison between groups was analyzed using one-way ANOVA. In addition, the frequency and proportion were presented for a diagnosis of shoulder disease, the number of treatments, the type of examination, and severity. In order to examine the characteristics of police officers complaining of back pain, the frequency of pain complaints by job function was investigated. The pain and function were expressed as the mean and standard deviation using the ODI, and the severity was investigated as the frequency and proportion using the classification of ODI scores. To examine the degree of shoulder and back pain and occupational factors, the frequency and proportion were classified according to the NIOSH standards, and cross-analysis was performed to confirm significant differences between shoulder and back pain using chi-square tests.

## 3. Results

In total, 9421 police officers participated in the survey, agreeing with its purpose. Among them, 4314 police officers completed all the survey questions. Public respondents included 2533 individuals who participated in Korea’s 2020 National Health and Nutrition Examination Survey. The survey included 3689 male and 625 female police officers, and among the civilians, there were 1309 men and 976 women. There was a statistically significant difference in the sex distribution between police officers and the general public. This is likely due to the higher representation of men in the police profession. There were no significant differences in age or cohabitation status between police officers and general public.

In terms of work patterns, 1945 police officers engaged in regular day–night shifts, whereas among the general public, 97 individuals followed the same work pattern. Additionally, 215 police officers and 14 individuals from the general public worked 24 h shifts. Furthermore, 381 police officers and 20 individuals from the general public were engaged in irregular shift work. Police officers had significantly longer working hours than individuals in the general public. There were also statistically significant differences in work patterns, with police officers having a relatively higher proportion of shift work (day–night and 24-h shifts) (Table 1).

When examining the work activity characteristics of civilians and police officers, the general public had a 2.3% rate of engaging in high-intensity work activities (physical activities causing heavy breathing or a rapid heart rate for more than 10 min), whereas police officers had a rate of 38.8%. The proportion of police officers engaging in high-intensity work activities was significantly higher than that of the general public, with police officers being approximately 17 times more likely to be exposed to high-intensity work activities than the general public (OR: 17.06). Furthermore, for moderate-intensity work activities (physical activities causing slight shortness of breath or a slightly faster heart rate for more than 10 min), the general public had a rate of 10.4%, whereas police officers had a rate of 47.3%. Police officers were approximately 4.5 times more likely to be exposed to moderate-intensity work activities than the general public (OR: 4.59) (Table 2).

To assess the regular physical activity of the general public and police officers, we surveyed the number of days they engaged in walking and strength training per week. There was a statistically significant difference in the frequency of walking for more than 10 min per week between the general public and police officers. Specifically, among those who did not walk for more than 10 min within a week, 3.1% were police officers and 17.4% were the general public. Furthermore, the proportion of individuals who walked more than 3 days a week was lower for the general public (63.3%) than for police officers (78.4%). There was also a significant difference in the number of strength training days. The percentages of police officers and the general public who did not engage in strength training were 34.9% and 71.5%, respectively (Table 3).

An investigation into the work functions of police officers who experienced musculoskeletal pain revealed nine significant factors. These factors included wearing police equipment, apprehending suspects, prolonged driving of police vehicles, extended investigative work, conflicts with demonstrators, wearing protective gear during protests, high-intensity training for protest management, traffic control duty, and extended motorcycle driving by police officers. The ranking of the aggravating factors in terms of prevalence was as follows: wearing police equipment at 86.5%, apprehending suspects at 79.8%, and prolonged driving of police vehicles at 76.4%. Among police officers, those working at local police stations and precincts experienced these aggravating factors most frequently. Police officers at local police stations and precincts reported wearing police equipment at a rate of 95.27%, apprehending suspects at a rate of 94.09%, and prolonged driving of police vehicles at a rate of 87.09% (Table 4).

All nine factors accounted for >40% of the overall prevalence of musculoskeletal pain. Prolonged investigative work (60.7%) and constant wearing of police equipment (64.5%) were associated with the highest prevalence rates of triggering musculoskeletal pain. When assessing pain intensity using the 100 mm pain VAS in cases in which pain was induced, conflicts with demonstrators and traffic control duties received the highest scores at 53 points. Following that, prolonged investigative work and apprehending suspects were noted (Table 5).

To assess shoulder pain and function in police officers who had experienced shoulder pain, four groups were created based on whether they had experienced pain in the past year or month. The number of those who reported experiencing pain in both the past year and the past month (referred to as YY) was 1601, accounting for 35.33% of the total. Using the SPADI to assess pain and functional impairment, a significant difference in functional impairment among the four groups was found. However, in terms of pain intensity, there was no significant difference between the group that experienced pain in the past year but not in the past month (NY) and the group that did not experience pain in the past year but had in the past month (YN). In terms of range of motion (ROM), the YY group exhibited a higher rate of functional impairment than the other groups, with the highest rates observed for flexion and forward flexion (FF), abduction (Abd), and internal rotation and adduction of the shoulder back (Irb) movements (Table 6).

Using the SPADI score, an investigation into shoulder disorders among police officers based on their work functions revealed scores ranging from a minimum of 16.28 to a maximum of 26.17. Scores were highest in the areas of traffic investigation, community safety, and traffic safety, in that order (Table 7). Significant differences in SPADI scores by job function were observed, with traffic investigation, community safety, and traffic safety showing differences compared with mobile police companies (comparison of police officers by job function using one-way ANOVA).

In an evaluation of the SPADI scores for police officers who experienced shoulder pain in the past year and within the past month, the scores were highest for traffic investigation (42.37), public safety (40.87), and the comprehensive civil complaints office (38.49), in that order. Police officers who had experienced shoulder pain within the past year and month generally had higher SPADI scores than those who had experienced shoulder pain (Table 8).

A survey on medical utilization was conducted to assess the management status of police officers who complained of shoulder pain. Of the 4314 total participants, 1445 (33.5%) reported visiting a health care facility due to shoulder pain. Among 1445 visitors to medical institutions, cases diagnosed with muscle pain accounted for the highest proportion at 445 (30.8%). Following that, the diagnostic rates were highest for rotator cuff disorders at 16.61%, followed by shoulder impingement at 13.43%, and adhesive capsulitis at 12.18%. These are stages progressing into degenerative shoulder conditions. Additionally, 11.42% of patients were diagnosed with rotator cuff tears, a condition characterized by irreversible degenerative changes in the shoulder that may require surgical treatment (Table 9).

According to the survey on the examination and treatment of shoulder pain among police officers, the majority, 51.56%, had received treatment less than 10 times. Additionally, for pain examinations, simple radiological testing such as X-ray imaging accounted for 64.01% of cases. For the diagnosis of other potential issues, such as tears and neurological damage, CT, MRI, and ultrasound examinations were reported at rates of 25.19%, 21.04%, and 33.29%, respectively (Table 10).

Out of the 1445 individuals who complained of shoulder pain, 469 (32.46%) received a diagnosis of tendon and muscle tears, with 255 (54.37%) being referred for surgical treatment. Among patients who required surgery, 158 (61.96%) underwent the procedure. Approximately 77.39% of police officers diagnosed with tears are currently experiencing the condition, but only 51.17% have undergone treatment (Table 11).

The results of comparing lumbar disability and pain intensity among police officers based on their job functions using the ODI scores revealed that the community safety work function had the highest score at 15.69, followed by police administration and human resources with 12.36 points, and the 112 emergency dispatch center with 11.91 points (Table 12).

The severity of lower back pain among police officers was investigated based on the ODI score classification by job function. Job functions with a high percentage of moderate to severe lower back pain (an ODI score of 21 or higher) were as follows: community safety (29.1%), traffic investigation (22.2%), police administration (19.8%), and the 112 emergency dispatch center (19.6%) (Table 13). In terms of ODI scores, statistically significant differences were observed when compared with the community safety function, particularly in the police precinct/box, investigation (sophisticated/economic/cyber), detective (major, violent, narcotic), and mobile police company functions (comparison of police officers by job function using one-way ANOVA).

Surgical treatment was performed on 17.83% of the police officers who experienced lower back pain. Among police officers who reported lower back pain, 63.32% had a persisting condition, while the treatment rate specifically for lower back pain was 36.37% (Table 14).

To evaluate the degree of occupational factors related to common musculoskeletal pain, such as shoulder and lower back pain, a survey was conducted to assess pain and frequency, classified according to the NIOSH standards. The results of the survey on the prevalence of musculoskeletal symptoms among police officers in the past year showed that the proportion of those who experienced shoulder pain was 54.41%, whereas that of those who experienced lower back pain was 57.14%, both exceeding half of the respondents. According to the NIOSH classification to assess pain intensity, the proportion of patients in Stage 2 or higher (Stage 2, symptoms persisting for more than a week, occurring at least once a month with moderate pain; Stage 3, symptoms persisting for more than a week, occurring at least once a month with severe pain) was 38.41% for shoulder pain and 41.34% for lower back pain (Table 15).

In musculoskeletal diseases, all joints such as the neck, shoulder, waist, knee, and ankle have a mutual mechanical relationship based on the spine. According to whether police officers had experienced shoulder pain in the past year, classification of the ODI scores was conducted. The results showed that among police officers who had experienced shoulder pain, the proportion of those with moderate to severe functional impairment was 25.4%, which was significantly higher than the 8.5% rate among those who had not experienced shoulder pain (Table 16).

## 4. Discussion

This study was conducted using a survey of current police officers to examine the status of musculoskeletal disorders according to job characteristics. This study aimed to collect information for the development of a system to prevent and manage musculoskeletal disorders related to police officers’ jobs in the future.

In terms of the general characteristics of the study participants, police officers had longer average working hours than civilians; regarding work patterns, police officers had a higher prevalence of rotating day–night shifts and irregular shifts. By contrast, civilians predominantly had daytime and evening work patterns. Police officers’ relatively irregular work patterns, stemming from the nature of their profession, result in irregularities in their daily routines. This can affect sleep patterns and disrupt circadian rhythms, leading to excessive tension in the musculoskeletal system. Several studies have also shown that shift work causes sleep and digestive disorders due to imbalanced biological rhythms. In addition, it worsens health conditions by causing negative behavioral changes such as persistent fatigue and irritability, which do not disappear even after rest [28]. The South Korean National Metropolitan Police employs a work system consisting of four teams with two shifts each, operating during the day from 09:00 to 21:00 and at night from 21:00 to the next day at 09:00 [29]. On a year-round basis, reports during night and late-night hours rapidly increase during summer holidays, the year’s end, New Year holidays, and various holiday periods. However, the four-shift work system does not guarantee sufficient rest time. In the case of night shifts, although there are 3 h of consecutive work followed by 1 h of a break within the 12 h work period, it is difficult to normalize the body’s rhythm in this format. Such work patterns are closely related to the health of police officers, especially irregular work patterns, which can continuously induce excessive muscular tension and exacerbate musculoskeletal disorders.

The investigation of the work intensity and daily physical activity of police officers and the general population revealed that police officers engaged in significantly higher proportions of high-intensity work activities and strength exercises than the general population. The average duration and frequency of exercise recommended by the World Health Organization, the American Heart Association, and the Centers for Disease Control and Prevention are approximately 150 min of aerobic exercise per week. However, for police officers, the exercise time was higher than the average recommended, which can be attributed to the high rate of high-intensity work activities among police officers. According to several studies, moderate exercise reduces physical fatigue and increases physical activity [30] but high-intensity work and the high rate of strength training of current police officers can cause hyperactivity of the musculoskeletal system, resulting in considerable physical fatigue [31].

Factors that aggravate the musculoskeletal system of police officers include wearing police equipment (firearms and radios), arresting criminals in the act, driving police vehicles for long periods of time, investigating for long periods of time, clashes with demonstrators, wearing protective gear for rallies, high-intensity training in preparation for rallies, working with traffic guidance hand signals, and long-term motorcycle driving. These aggravating factors account for a large proportion of work activities in the district police boxes. Through this, it was found that the work activities of the district police stations had a significant impact on the deterioration of the musculoskeletal system. Furthermore, when assessing the intensity of pain caused by the nine aggravating factors for musculoskeletal issues, most cases showed a VAS score of 50 or higher, which typically indicates the need for professional treatment. Given that the VAS scores for the nine musculoskeletal exacerbating factors mostly exceeded 50 points, this indicated that many cases require professional treatment, although they may not necessarily disrupt daily life or work to an extent that prevents functioning. This indicates the need to establish a systematic management system through comprehensive research and support because there is a high likelihood that musculoskeletal disorders among police officers can develop into serious conditions when appropriate preventive measures and management are lacking. Additionally, pain scores were the highest for duties involving clashes with protest groups and traffic control duties. The two aforementioned types of work involve repetitive movements. This appears to have resulted in high pain scores owing to muscle overuse, which is the most important cause of musculoskeletal disorders.

In a study involving police officers who experienced shoulder pain, the SPADI and ROM were examined. The results showed that in the group of police officers who had shoulder pain within the past year and within the past month (YY), the number of cases with ROM impaired by more than 50% was significantly higher than that in the other groups. This suggests that among individuals with chronic musculoskeletal disorders, those who currently experience pain face the most significant functional impairments. In other words, among police officers who chronically complain of musculoskeletal pain, an intensive management system is needed for those who have most recently complained of pain. Shoulder pain has different patterns depending on the disease; however, in the case of adhesive capsulitis, pain gradually increases, people complain of nighttime pain, and the pain worsens from the first stage of inflammation to the second stage of freezing. In contrast, calcific tendinitis presents with severe pain and a limited range of motion as the calcium deposits are absorbed in the resorptive phase [32]. Therefore, even if pain has recently occurred, it is essential to accurately confirm the diagnosis. In particular, police officers who have recently complained of pain require professional treatment. Additionally, officers who experienced pain within the most recent year or month had higher overall SPADI scores. This also revealed that the most recent onset of pain was associated with a higher pain intensity. By type of work, it was found that the SPADI scores were the highest in the order of traffic surveys and community safety. This suggests that improvement measures should be prepared by checking whether there are harmful factors that may affect police officers’ shoulder disease in the two work types above.

The investigation into the diagnosis and treatment of shoulder pain among police officers revealed that 33.5% of all surveyed police officers had visited a medical institution for shoulder pain. This proportion was lower than the overall percentage of police officers who reported shoulder pain, indicating that a significant number of individuals with musculoskeletal pain did not seek medical diagnosis. The low rate of visits to medical institutions indicates a lack of connection between pain and treatment, which should be improved by identifying the cause through a systematic investigation. Furthermore, among police officers who visited medical institutions for a diagnosis, 6.57% had never received treatment and more than half (51.56%) had received treatment fewer than 10 times. Typically, for simple shoulder pain, the treatment period is at least 2 weeks. In the case of a rotator cuff tear, a partial tear may require 3–6 months of conservative treatment, and if surgery is necessary, treatment including rehabilitation for more than a year may be required [33]. However, the majority of police officers received less than 10 treatments, which means they were not treated properly. The proportion of police officers diagnosed with shoulder tendon muscle rupture was 32.46%. However, more than half (64.01%) of the tests conducted at medical institutions were X-rays; therefore, it is possible that there was actually a rupture but it might not have been diagnosed because it was not accompanied by an ultrasound, CT, or MRI diagnosis. Among police officers diagnosed with a shoulder tendon rupture, more than half (61.96%) underwent surgery; however, 77.39% reported that the disease persisted. This suggests that satisfaction with surgical treatment is low, as additional treatments such as rehabilitation are not provided after surgery. In summary, there is a significant lack of satisfaction with surgical treatment, and a substantial proportion of police officers with shoulder pain do not receive a proper diagnosis or ongoing treatment. This highlights a deficiency in the system and alternatives for managing the musculoskeletal health of police officers.

Using the ODI, the severity of lower back pain among police officers was investigated on the basis of their job functions. The results showed that job functions with a high proportion of moderate to severe scores, classified by an ODI score criterion of 20 or higher, were those with high levels of physical activity, such as community safety, traffic investigation, and the 112 emergency room. The frequency of back pain complaints due to work function was significantly higher in district police stations. According to the ODI score classification criterion, a score of 20 or higher indicates the presence of functional disability accompanying back pain. Degenerative changes in the spine typically begin to appear in the late teens or early twenties. These changes result from aging of the intervertebral discs, which can lead to the development of herniated discs when subjected to pressure, especially in situations involving excessive bending or twisting while handling heavy objects. Of course, not all cases of back pain require surgery, and surgery is considered when there is no response to conservative treatment lasting for over 6 months or when there is persistent leg pain lasting for more than 3 months [34]. However, according to a survey, while 63.32% of police officers reported experiencing back pain, only 36.37% of those who complained of pain underwent treatment, and only 17.83% underwent surgical treatment. Treatment of back pain typically requires long-term conservative care and postoperative management. Therefore, the low treatment rates indicated by the survey results highlight the urgent need to improve the management of musculoskeletal disorders among police officers.

The cross-analysis of shoulder and back pain revealed that more than half of the surveyed police officers who responded had experienced both shoulder and back pain within the past year. When assessing the severity of each condition, shoulder pain scored 51 points and back pain scored 50 points, indicating the need for expert treatment in both cases. In particular, the results of the investigation using NIOSH’s musculoskeletal disorder classification criteria revealed that situations requiring caution at the second stage or above (symptoms persisting for more than one week and experiencing intermediate pain at least once a month) were confirmed to be approximately 38.41% for the shoulder and 41.34% for the lower back. This indicates that nearly 40% of the cases require a management intervention.

The cross-analysis of shoulder and back pain revealed that 25.5% of police officers with shoulder pain had moderate to severe back pain, indicating the occurrence of musculoskeletal pain in multiple areas among active-duty police officers. Several studies have shown that prolonged equipment wear is strongly correlated with musculoskeletal pain in multiple areas [35]. In particular, symptoms of tension, such as an increased heart rate, were observed when holding a load weighing 10.4 kg in stationary and standing positions [36]. In the case of firefighters, protective gear weighs approximately 25 kg, which puts strain on the body. Police officers can also have an impact on their bodies by wearing heavy gear. In fact, a police officer’s bulletproof vest weighs 2.9 kg, and when equipment such as firearms, handcuffs, radios, and truncheons is added, the final weight has a negative effect on the musculoskeletal system [37]. Moreover, as shown by the results indicating that the health-related quality of life is significantly lower in individuals with both neck and shoulder pain and lower back pain compared with those without such pain, various musculoskeletal disorders in multiple body parts of police officers caused by prolonged equipment use can impact not only the health of police officers but also their overall lives. This situation, directly linked to the safety of the public, underscores the necessity for systematic management through established protocols.

Despite the results above, this study has limitations as a cross-sectional study using a survey. First, it has limitations in directly proving causal relationships. Second, there is a disadvantage that not everyone could access the online survey, and there are also concerns about the security and protection of information. However, this study conducted a collaborative study with the National Police Agency of the Republic of Korea to ensure that all police officers could access the survey study. Third, this study has limitations in that it did not cover all aspects of the phenomena that occur, and the scope of the questions was limited. Finally, the results may be biased because the sample did not accurately reflect the population

## 5. Conclusions

This study surveyed police officers in South Korea to investigate status of musculoskeletal disorders according to job characteristics. The study found no statistically significant differences in general characteristics between police officers and the general population. However, significant differences were observed in work patterns and exercise intensity, with police officers showing higher values. Among the police officers’ work types, nine factors that deteriorate the musculoskeletal system were identified. These factors could be identified repeatedly in the type of work of police officers complaining of shoulder and back pain. This suggests that police officers are at a higher risk of developing musculoskeletal disorders owing to their occupational characteristics. This study revealed a low treatment rate among police officers who complained of musculoskeletal pain. Although the job type and environment of police officers directly affect musculoskeletal diseases, these can occur as degenerative diseases caused by aging, making it difficult to assess their job relevance. Therefore, it is necessary to establish a systematic management and tracking system for well-executed studies on the musculoskeletal health of police officers. To activate a system that can continuously manage and prevent the health problems of police officers, national police-related organizations (such as the National Police Agency, the National Police University, and the National Police Hospital) and medical data-oriented research hospitals must cooperate to establish a medical data management system for police officers.

## Figures and Tables

**Table 1:** General characteristics of the study participants.

Variables		General Public	Police Officers	*p*
Total		2533	4314	
Sex (n)	Female	1224	625	<0.001
	Male	1309	3689	
Age (years)	Mean ± SD	43.9 ± 10.2	43.7 ± 10.0	0.4278
Presence of a cohabitant (n)	No	254	527	0.0332
	Yes	2279	3787	
Work hours	Mean ± SD	40.6 ± 14.5	46.6 ± 17.3	<0.001
Work arrangement (n)	Daytime shift	2125	1388	<0.001
	Night shift	222	20	
	Overnight shift	50	64	
	Regular rotating day and night shift work	97	1945	
	24 h rotating shift work	14	215	
	Split shift	5	26	
	Irregular rotating shift work	20	381	
	Other	-	275	

SD, standard deviation. Nighttime shift: 2:00 p.m. to 12:00 a.m.; nighttime shift: 9:00 p.m. to 9:00 a.m.; split shift: more than two work periods within a day.

**Table 2:** Comparison of the ratios for the intensity of work activity among the study participants.

Intensity of Work Activity		General Public (n = 2533)	Police Officers (n = 4268)	χ^2^ (*p*)	OR (95% CI)
High-intensity work activities	Yes	58 (2.3%)	1655 (38.8%)	<0.001	17.06 (13.15–22.14)
	No	2475 (97.7%)	2613 (61.2%)		
Moderate-intensity work activities	Yes	263 (10.4%)	2019 (47.3%)	<0.001	4.59 (4.04–5.21)
	No	2270 (89.6%)	2249 (52.7%)		

OR, odds ratio; CI, confidence interval; Y, yes; N, no. High-intensity work activities: physical activities causing heavy breathing or a rapid heart rate for more than 10 min; moderate-intensity work activities: physical activities causing slight shortness of breath or a slightly faster heart rate for more than 10 min.

**Table 3:** Number of days of walking and strength training among the study participants.

Categorization	General Public (n = 2533)	Police Officers (n = 4268)	χ^2^ (*p*)
Number of days of walking in the last week (walking for 10 min or more)			
None	442 (17.4%)	133 (3.1%)	<0.001
1 day	205 (8.1%)	206 (6.1%)	
2 days	285 (11.3%)	529 (12.4%)	
3 days	311 (12.3%)	848 (19.9%)	
4 days	172 (6.8%)	522 (12.2%)	
5 days	331 (13.1%)	628 (14.7%)	
6 days	124 (4.9%)	176 (4.1%)	
7 days	663 (26.2%)	1172 (27.5%)	
Numbers of days of strength training in the last week			
None	1181 (71.5%)	1490 (34.9%)	<0.001
1 day	118 (4.7%)	627 (14.7%)	
2 days	166 (6.6%)	727 (17.0%)	
3 days	181 (7.1%)	747 (17.5%)	
4 days	72 (2.8%)	310 (7.3%)	
More than 5 days	185 (7.3%)	367 (8.6%)	

**Table 4:** Factors related to musculoskeletal overuse in the shoulders.

Musculoskeletal Exacerbating Factors	Incident Experience Status			Work Function (Multiple Responses Allowed)
	Frequency	Total	Ratio (%)	
Long-term driving of police vehicles	3777	4944	76.4	Police precinct/box (87.09%) Traffic safety (16.38%) Detective (16.27%)
Long-term driving of police motorcycles	472	4911	9.61	Traffic safety (49.14%)
Wearing police gear (firearms, radio equipment)	4238	4898	86.5	Police precinct/box (95.27%) Traffic safety (10.83%) Detective (9.31%)
Arrest of a suspect caught in the act	3886	4866	79.8	Police precinct/box (94.09%) Detective (15.02%) Security (7.05%)
High-intensity training for protest and demonstration preparedness	2147	4827	44.4	Mobile police company (79.92%)
Wearing protest or demonstration protective gear	2158	4800	44.9	Mobile police company (80.53%)
Confrontation with protesters	2.309	4784	48.2	Mobile police company (82.96%)
Long-term investigation work	2375	4756	49.9	Police precinct/box (39.06%) Detective (30.98%) Investigation (27.09%) Traffic investigation (15.55%) Juvenile and sex-based violence Investigation division (13.86%)
Traffic signal duty	1.752	4735	37.0	Police precinct/box (59.91%) Traffic safety (51.57%)

**Table 5:** Relationship between musculoskeletal aggravating factors, occurrence of pain, and pain intensity.

Musculoskeletal Exacerbating Factors	Cause of Pain			Pain Intensity 100 mm VAS
	Frequency	Total	Ratio (*%*)	
Long-term driving of police vehicles	1854	3739	49.4	47
Long-term driving of police motorcycles	200	466	42.9	49
Wearing police gear	2718	4221	64.5	50
Arrest of a suspect caught in the act	1758	3856	45.5	52
High-intensity training for protest and demonstration preparedness	869	2127	40.8	51
Wearing protest or demonstration protective gear	1.041	2.147	48.4	51
Confrontation with protesters	946	2295	41.2	53
Long-duration investigation work	1437	2366	60.7	52
Traffic signal duty	626	1736	36.0	53
Total	10,409	20,808	50.02	50.88

VAS, visual analog scale.

**Table 6:** Comparison using the shoulder pain and disability index among police officers who have experienced shoulder pain.

Presence of Shoulder Pain (in the Past 1 Year/in the Past 1 Month)						*p*
Group		YY	NY	YN	NN	
Number		1601	221	866	1843	
Ratio		35.33	4.88	19.11	40.67	
SPADI (mean ± SD)						
Pain index		40.45 ± 26.86	31.29 ± 23.98	30.18 ± 23.10	13.26 ± 18.24	<0.001
Post hoc test		a	b	b	c	
Disability index		26.28 ± 25.54	17.30 ± 20.23	10.05 ± 14.75	5.87 ± 13.12	<0.001
Post-hoc test		a	b	c	d	
SPADI total		34.81 ± 23.78	22.68 ± 20.32	17.79 ± 16.28	8.71 ± 14.00	<0.001
Post hoc test		a	b	c	d	
Range of motion (ROM)						
FF	Normal	909	140	653	1488	
	0–25%	330	32	101	131	
	25–50%	290	40	93	145	
	50–100%	72	9	19	79	
Abd	Normal	857	133	639	1478	
	0–25%	368	38	120	166	
	25–50%	353	47	97	164	
	50–100%	23	3	10	35	
Irb	Normal	922	144	653	1429	
	0–25%	537	64	171	320	
	25–50%	115	10	30	62	
	50–100%	27	3	12	32	

YY, occurrence of shoulder pain in the past 1 year and in the past 1 week; NY, no shoulder pain in the past 1 year, but shoulder pain in the past 1 month; YN, had shoulder pain in the past 1 year but no shoulder pain in the past 1 month; NN, no shoulder pain in the past 1 year or in the past 1 week; SPADI, shoulder pain and disability index; ROM, range of motion; FF, flexion and forward flexion; Abd, abduction; IRB, internal rotation and adduction of the shoulder back. Data are presented as means ± SD. a, b, c, d indicate statistical differences in the post hoc test after one-way ANOVA.

**Table 7:** Distribution of shoulder pain among police officers by job function using the shoulder pain and disability index score.

Job Function	SPADI	
	Number	Mean ± SD
Traffic investigation	105	26.17 ± 24.69
Community safety	224	24.17 ± 24.77
Traffic safety	227	22.54 ± 23.65
Investigation (sophisticated/economic/cyber)	323	21.51 ± 22.71
112 emergency dispatch center	190	21.40 ± 22.00
Intelligence/national security/foreign affairs	170	20.73 ± 22.06
Police administration and human resources	175	20.41 ± 21.81
Police precinct/box	2016	20.33 ± 21.93
Civil services center	41	20.26 ± 17.66
Detective (major, violent, narcotic)	208	19.69 ± 21.28
Juvenile and sex-based violence investigation division	161	19.17 ± 21.51
Forensic investigation	68	16.36 ± 20.26
Mobile police company	472	16.28 ± 18.95
Other	151	20.02 ± 20.61
Total	4531	

SPADI, shoulder pain and disability index. Data are presented as means ± SD.

**Table 8:** Comparison of shoulder pain using the shoulder pain and disability index among police officers who reported shoulder pain in the past 1 year and past 1 month.

Current Job Function	In the Past 1 Year and 1 Month			SPADI Mean ± SD
	Number	Total	Ratio (%)	
Traffic investigation	37	95	38.9	42.37 ± 24.32
Community safety	81	214	37.9	40.87 ± 26.84
Civil services center	13	39	33.3	38.49 ± 13.36
Investigation	114	305	37.4	36.22 ± 22.64
Traffic safety	92	209	44.0	35.19 ± 24.24
112 emergency dispatch center	67	179	37.4	34.50 ± 22.96
Detective	75	198	37.9	35.19 ± 23.89
Police precinct/box	704	1932	36.4	34.82 ± 23.95
Intelligence/national security/foreign affairs	47	161	29.2	34.73 ± 27.02
Mobile police company	115	446	25.8	34.00 ± 21.20
Police administration and human resource	57	168	33.9	32.20 ± 24.56
Juvenile and sex-based violence investigation division	61	157	38.9	29.44 ± 23.82
Forensic investigation	16	65	24.6	29.06 ± 19.52
Other	49	146	33.6	20.75 ± 22.13
Total	1528	4314	35.4	

SPADI, shoulder pain and disability index. Data are presented as means ± SD.

**Table 9:** Diagnosis of shoulder pain among police officers.

Diagnoses Related to Shoulder Pain	Number	Total	Ratio (%)
Common shoulder pain	445	1445	30.80
Rotator cuff disorder	240	1445	16.61
Shoulder tendonitis	194	1445	13.43
Adhesive capsulitis	176	1445	12.18
Rotator cuff tear	165	1445	11.42
Calcific tendinitis	119	1445	8.24
Cervical disc disease	40	1445	2.77
Articular cartilage tear or fracture	15	1445	1.04
Impingement syndrome	8	1445	0.55
Shoulder joint dislocation	8	1445	0.55
External impingement	5	1445	0.35
Arthritis	3	1445	0.21
Other conditions	125	1445	8.65

**Table 10:** Examination and treatment of shoulder pain among police officers.

Number of Treatments	Number	Total	Ratio (%)
No treatment received	95	1445	6.57
1–5 times	452	1445	31.28
5–10 times	293	1445	20.28
10–20 times	254	1445	17.58
20–50 times	204	1445	14.12
More than 50 times	147	1445	10.17
Methods of examination during treatment of shoulder pain			
X-ray	925	1445	64.01
CT	364	1445	25.19
Joint ultrasound	304	1445	21.04
Shoulder MRI	481	1445	33.29
Fever test/analysis	37	1445	2.56
Never received testing	160	1445	11.07

**Table 11:** Assessment of the severity of shoulder pain among police officers.

Severity of Shoulder Pain	Number	Total	Ratio (%)
Diagnosis of shoulder tendon muscle tearing	469	1445	32.46
Recommendation for surgical treatment	255	469	54.37
Surgical intervention performed	158	255	61.96
Persistence of the condition	363	469	77.39
Treatment status of the condition	240	469	51.17

**Table 12:** Indicators of lower back pain by job function among police officers.

Work Function	ODI	
	Number	Mean ± SD
Community safety	214	15.69 ± 15.94
Police administration and human resources	168	12.36 ± 15.05
112 emergency dispatch center	179	11.91 ± 11.95
Police precinct/box	1932	11.88 ± 12.75
Juvenile and sex-based violence investigation division	157	11.85 ± 12.39
Investigation (sophisticated/economic/cyber)	305	11.63 ± 13.80
Traffic safety	209	11.53 ± 11.80
Intelligence/national security/foreign affairs	161	11.20 ± 12.03
Traffic investigation	95	11.09 ± 11.07
Detective (major, violent, narcotic)	198	11.04 ± 11.92
Forensic investigation	65	9.95 ± 11.70
Civil services center	39	9.76 ± 7.67
Mobile police company	446	9.65 ± 12.50
Other	146	12.63 ± 13.45
Total	4314	

ODI, Oswestry disability index. Data are presented as means ± SD.

**Table 13:** Police officers’ work function according to the lumbar pain index.

Work Function	Classification of ODI Scores (Lumbar Function Scale)						Total
		Mild (0–20)	Moderate (21–40)	Severe (41–60)	Impairment (61–80)	Inability/ Exaggeration (81–100)	
112 emergency dispatch center	N	144	31	4	0	0	179
	Ratio (%)	80.3	17.4	2.2	0.0	0.0	100.0
Police administration	N	134	25	7	1	1	168
	Ratio (%)	80.2	14.4	4.2	0.6	0.6	100.0
Mobile police company	N	384	48	8	5	1	446
	Ratio (%)	86.0	10.9	1.8	1.1	0.2	100.0
Forensic investigation	N	53	10	2	0	0	65
	Ratio (%)	81.5	15.4	3.1	0.0	0.0	100.0
Traffic safety	N	173	28	8	0	0	209
	Ratio (%)	82.6	13.5	3.9	0.0	0.0	100.0
Traffic investigation	N	74	20	1	0	0	95
	Ratio (%)	77.9	21.1	1.1	0.0	0.0	100.0
Community safety	N	152	44	12	5	1	214
	Ratio (%)	71.0	20.5	5.7	2.4	0.5	100.0
Investigation	N	252	40	8	4	1	305
	Ratio (%)	82.6	13.1	2.6	1.3	0.3	100.0
Female sexual assault investigation	N	130	20	6	1	0	157
	Ratio (%)	82.7	12.8	3.8	0.6	0.0	100.0
Intelligence	N	133	24	3	1	0	161
	Ratio (%)	82.5	15.0	1.9	0.6	0.0	100.0
Civil services center	N	36	3	0	0	0	39
	Ratio (%)	91.7	8.3	0.0	0.0	0.0	100.0
Police precinct/box	N	1591	269	57	13	2	1932
	Ratio (%)	82.7	13.8	2.8	0.6	0.1	100.0
Detective	N	168	27	1	1	1	198
	Ratio (%)	84.6	13.8	0.5	0.5	0.5	100.0
Other	N	115	23	7	1	0	146
	Ratio (%)	79.7	14.7	4.9	0.7	0.0	100.0
Total	N	3508	602	121	30	7	4314
	Ratio (%)	82.2	14.1	2.8	0.7	0.2	100.0

N, number; ODI, Oswestry disability index.

**Table 14:** Severity of lower back pain among police officers.

Severity of Back-Related Pain	Number	Total	Ratio (%)
Surgical treatment performed	350	1963	17.83
Continuation of the condition	1243	1963	63.32
Treatment status of the condition	714	1963	36.37

**Table 15:** Police officers’ musculoskeletal symptom status and comparison using the visual analog scale and National Institute of Occupational Safety and Health classifications.

Location of the Symptoms’ Manifestation	Symptoms Occurring within the Past Year (Pain, Stiffness, etc.)			VAS	NIOSH Classification		
	Number	Total	Ratio (%)		Stage 1	Stage 2	Stage 3
Shoulder	2558	4701	54.41	51	1562	640	334
					61.59%	25.24%	13.17%
Lower back	2528	4.424	57.14	45	1471	681	356
					58.65%	27.15%	14.19%

VAS, visual analog scale; NIOSH, National Institute of Occupational Safety and Health.

**Table 16:** Cross-analysis of shoulder and back pain among police officers.

Presence of Shoulder Pain		Classification of ODI Scores (Lumbar Function Scale)					Total
		Mild	Moderate	Severe	Impairment	Inability/ Exaggeration	
No	N	1775	116	31	17	5	1944
	Ratio (%)	91.3	6.0	1.6	0.9	0.3	100.0
Yes	N	1733	486	90	13	2	2324
	Ratio (%)	74.6	20.9	3.9	0.6	0.1	100.0
Total	N	3508	602	121	30	7	4268
	Ratio (%)	82.2	14.1	2.8	0.7	0.2	100.0

N, number; ODI, Oswestry disability index.

## Data Availability

The raw data supporting the conclusions of this article will be made available by the authors on request.
